# Selenium Compound Protects Corneal Epithelium against Oxidative Stress

**DOI:** 10.1371/journal.pone.0045612

**Published:** 2012-09-25

**Authors:** Akihiro Higuchi, Hiroyoshi Inoue, Tetsuya Kawakita, Tadashi Ogishima, Kazuo Tsubota

**Affiliations:** 1 Shinanomachi Research Park 6N9, Center for Integrated Medical Research, Keio University, School of Medicine, Tokyo, Japan; 2 Department of Ophthalmology, Keio University, School of Medicine, Tokyo, Japan; 3 Department of Chemistry, Keio University, School of Medicine, Kanagawa, Japan; 4 Department of Chemistry, Faculty of Sciences, Kyushu University, Fukuoka, Japan; National Cancer Institute, United States of America

## Abstract

The ocular surface is strongly affected by oxidative stress, and anti-oxidative systems are maintained in corneal epithelial cells and tear fluid. Dry eye is recognized as an oxidative stress-induced disease. Selenium compound eye drops are expected to be a candidate for the treatment of dry eye. We estimated the efficacy of several selenium compounds in the treatment of dry eye using a dry eye rat model. All of the studied selenium compounds were uptaken into corneal epithelial cells *in vitro*. However, when the selenium compounds were administered as eye drops in the dry eye rat model, most of the selenium compounds did not show effectiveness except for Se-lactoferrin. Se-lactoferrin is a lactoferrin that we prepared that binds selenium instead of iron. Se-lactoferrin eye drops suppressed the up-regulated expression of heme oxygenase-1, cyclooxygenase-2, matrix metallopeptidase-9, and interleukin-6 and also suppressed 8-OHdG production in the cornea induced by surgical removal of the lacrimal glands. Compared with Se-lactoferrin, apolactoferrin eye drops weakly improved dry eye in high dose. The effect of Se-lactoferrin eye drops on dry eye is possibly due to the effect of selenium and also the effect of apolactoferrin. Se-lactoferrin is a candidate for the treatment of dry eye via regulation of oxidative stress in the corneal epithelium.

## Introduction

Selenium is an essential trace element for animals. Selenium is a component of the amino acid selenocysteine (Sec**;** U), which is a cysteine analogue with a selenium atom replacing a sulfur atom. Proteins containing Sec are called selenoproteins. Twenty-five selenoprotein genes are present in the human genome [1], but only a few of these proteins have been functionally characterized, *e.g.* **,** glutathione peroxidases (GPx), thioredoxin reductases (TrxR), and iodothyronine deiodinases (DIO), which all have oxidoreductase functions [2]. GPx and TrxR participate in the reduction of hydrogen peroxide and lipoperoxide [2], [3]; therefore, the physiological role of GPx and TrxR is regulation of oxidative stress. Since the active site of GPx and TrxR contains the Sec residue, selenium is essential for the activity of these enzymes [3].

Ocular surface cells are strongly affected by oxidative stress caused by several factors, *e.g.*, light exposure including ultraviolet (UV) irradiation [4] and direct contact with airflow [5] and chemical compounds [6]. To protect the corneal epithelium against oxidative stress from outside, antioxidative enzymes are expressed in corneal epithelial cells [7]. GPx is widely distributed in tissues of the body including the ocular surface [7], [8]. Since GPx is expressed in corneal epithelial cells, a steady supply of selenium for corneal epithelial cells is required to maintain the enzyme activity of GPx in the corneal epithelium. Selenoprotein P (SeP) is known as a selenium-transfer plasma glycoprotein [9] and is present in extracellular fluids such as plasma [10] and milk [11]. Our previous study showed that SeP was expressed in lacrimal glands and secreted in tear fluid to supply selenium to the corneal epithelium, and the SeP concentration in tear fluid was reduced in dry eye patients [12]. Furthermore, because of the shortage of selenium from the lacrimal glands in dry eye patients, corneal damage was induced accompanied by an increase in oxidative stress in the cornea. Because SeP eye drops rescued this corneal damage, we concluded that SeP was useful for the treatment of dry eye [12]. Although SeP was a good candidate for clinical application to the treatment of dry eye, it is difficult to synthesize large amounts of SeP using cultured systems.

Tear fluid contains many kinds of anti-oxidative stress compounds such as vitamin C, glutathione, superoxide dismutase, and lactoferrin [13], [14]. Lactoferrin also protects the corneal epithelium against UV irradiation [15]. Previous studies demonstrated that the concentration of lacrimal lactoferrin was reduced [16] and oral administration of lactoferrin improved symptoms in dry eye patients with Sjogren’s syndrome [17].

Lactoferrin is an iron-binding glycoprotein and is found in most exocrine fluids such as saliva, bile, pancreatic fluid, amniotic fluid and tears [18]. The most common metal ion associated with lactoferrin in vivo is iron in its ferric (III ) form. Lactoferrin can also bind other metal ions such as copper and magnesium. We could successfully prepare selenium-binding lactoferrin (Se-lactoferrin) in the present study. Se-lactoferrin is expected to have two effects, i.e., as an anti-oxidative stress compound and as a selenium supplier to the ocular surface. In this study, we investigated new candidates for the treatment of dry eye from several selenium compounds.

## Materials and Methods

### Preparation of Se-lactoferrin

We initially produced iron-free apolactoferrin from bovine milk using the UF-membrane system with citric acid [19]. One g of apolactoferrin and 5.4 mg of selenium (I ) chloride were dissolved in 10 mL of deionized water. The solution was stirred at 4°C for 60 min. The solution was filtered three times using the UF filtration apparatus (FPS-24001, Asahi Kasei Corporation, Tokyo, Japan) with a MW 6000-cut membrane filter. The filtered solution was then lyophilized. The apolactoferrin powder was dissolved again with deionized water and was adjusted to pH 1.0 with 0.1 M HCl. The selenium content in Se-lactoferrin was determined with 2,3-diaminonaphthalene using a fluorometer (FP6000, JASCO Corporation, Hachioji, Tokyo, Japan) [20]. As a control, normal bovine milk lactoferrin, which binds approximately 20% iron in molar ratio, was used.

### Uptake of Selenium Compounds using Corneal Epithelial Cells

The effect of selenium compounds on cellular viability was estimated using the human corneal epithelial cell line, CEPI-17-CL4 (CEPI) cells [21]. CEPI cells were cultured to 80–90% confluence in medium (Epilife, Cascade Biologics Inc., Portland, OR, USA) supplemented with HCGS (Cascade Biologics Inc.) in 96-well plates. Selenium compounds, *i.e.*, sodium selenite, Sec, selenomethionine (SeMet), Sec-containing peptides (Peptide-1 and Peptide-2), and Se-lactoferrin, were added to the medium and CEPI cells were incubated for 24 hours. The amino acid sequence of Peptide-1 was RSUS and that of Peptide-2 was RSUSSHSRHLIFEK. Peptides were synthesized by the Fmoc method. Sec, SeMet, Peptide-1, and Peptide-2 each contain one atom of selenium.

The medium was changed to fresh medium containing 10% Alamar Blue (Wako Pure Chemical Industries, Ltd., Osaka, Japan), which is a quantitative reagent used to evaluate cellular viability. After 1 hour incubation, the fluorescence of Alamar Blue was measured by a multilabel reader (ARVO SX; PerkinElmer Japan Co., Ltd., Yokohama, Kanagawa, Japan). The selenium concentration from each selenium compound in the medium was 0, 0.16, 1.25, 5, or 20 µM, respectively.

The efficiency of selenium uptake to CEPI cells from selenium compounds was estimated using selenium-deprived CEPI cells as described in our previous study [12]. CEPI cells were cultured in EpiLife with (+) or without selenium (−). Selenium deficiency was monitored every week by measuring GPx activity in the cell lysate. GPx is a well-characterized selenoprotein, in which the selenium atom is essential to catalyze the enzyme reaction. After GPx activity diminished, selenium compounds were added to the medium and the cells were incubated for 24 hours. If selenium was uptaken into CEPI cells, GPx activity was recovered. The selenium concentration from each selenium compound in the medium was 0, 0.005, 0.05, 0.5, or 5 µM, respectively. The GPx activity ratio was calculated as the ratio of the activity level of CEPI cells cultured with a selenium compound to the activity level of CEPI cells cultured in EpiLife with selenium. The activity level of CEPI cells cultured in EpiLife with selenium was defined as 1.

### Effects of Selenium Compounds on Treatment for Dry Eye using a Rat Model

All animal experiments were approved by the Laboratory Animal Care and Use Committee of Keio University School of Medicine. The effect of selenium compounds on the treatment of dry eye was estimated by applying selenium compound eye drops to the dry eye rat model. Six-week-old, male Sprague-Dawley rats were purchased from CLEA Japan, Inc. (Tokyo, Japan). The dry eye rat model was prepared by removing the lacrimal glands from anesthetized rats [12], [22]. The fluorescein score, which is an index of dry eye, was estimated as described in our previous study [12]. To estimate the degree of dry eye condition in the cornea, the ocular surfaces of rats were photographed after being stained by fluorescein for scoring as follows. The photographs of the corneas were divided into 9 areas. Each area was scored from 0 to 3 points depending on the degree of staining, and the scores of all areas were totaled. This total was called the ‘fluorescein score’. One eye in each dry eye rat was treated with a selenium compound and the other eye was treated with phosphate-buffered saline (PBS) (n = 10 in each group). 0.01, 0.1, or 1% Se-lactoferrin eye drops contained 1.8, 18, or 180µM selenium, respectively. Our previous study showed that PBS treatment did not suppress the increased fluorescein scores in dry eye rats [12]. Se-lactoferrin is thought to dissociate into a selenium ion and apolactoferrin in PBS. Considering the influence of apolactoferrin in the treatment of dry eye, apolactoferrin was used as the control drug instead of PBS when 0.1% Se-lactoferrin eye drops were used. Five µL of each eye drops was administered 4 times per day for 2 weeks. After all eye drop treatments, the corneal fluorescein scores were estimated, and the corneas were collected to isolate RNA for real-time PCR, or the bulb of eyes was collected for use in the immunohistochemical study.

### Effect of Se-lactoferrin Eye Drops on Gene Expression in Rat Corneas

Corneas were removed from the eyeballs of normal rats (NT), dry eye rats treated with PBS (PBS), and dry eye rats treated with 0.1% Se-lactoferrin (0.1% Se-lactoferrin). Total RNA was extracted from corneas using TRIzol (Life Technologies Corporation, Carlsbad, CA, USA) for quantitative analysis of gene expression. Reverse transcription (RT) was performed using SuperScript III (Life Technologies Corporation) according to the manufacturer’s protocol. Gene expression of SeP, GPx-1, tumor necrosis factor-α (TNF-α), androgen receptor (AR), estrogen receptor (ER), heme oxigenase-1 (HO-1), cyclooxygenase-2 (COX-2), matrix metallopeptidase (MMP)-2, MMP-9, or interleukin-6 (IL-6) was estimated by TaqMan real-time RT-PCR (ABI PRISM 7500 Sequence Detection Systems, Life Technologies Corporation) according to the manufacturer’s protocol. Primer sets and TaqMan probes, and other reagents for TaqMan real-time PCR were purchased from Life Technologies Corporation. All data were analyzed with the ΔΔCt method and the mRNA of glyceraldehyde 3-phosphate dehydrogenase (GAPDH) was used as the internal standard.

### Immunohistochemical Analysis of an Oxidative Stress Marker in Corneas

To estimate the level of oxidative stress markers in corneas, immunohistochemical analysis was performed using anti-8-hydroxy-2′-deoxyguanosine (8-OHdG) antibody according to the manufacturer’s protocol. 8-OHdG is a marker of oxidative DNA damage and most suitable oxidative stress marker in cornea [12], [23]. Briefly, eyeballs removed from normal rats, dry eye rats, dry eye rats treated with PBS, and dry eye rats treated with 0.1% Se-apolactoferrin were fixed with 10% neutral buffered formalin and embedded in paraffin. Five-µm thick sections were deparaffinized, rehydrated, and heated in an autoclave for 10 minutes in citrate buffer (pH 6.0) for antigen retrieval. Nonspecific binding was inhibited by incubating the specimens with 10% normal goat serum (Life Technologies Corporation) at room temperature for 30 minutes. The specimens were incubated with the optimally diluted primary antibody at 4°C overnight, followed by incubation with a peroxidase-conjugated goat anti-rabbit or anti-mouse IgG antibody (Histofine Simple Stain Max-PO, Nichirei Biosciences Inc., Tokyo, Japan) at room temperature for 45 minutes. 8-OHdG was visualized by staining with diaminobenzidine tetrahydrochloride. Staining of nuclei was simultaneously performed with hematoxylin. All steps were followed by three washes with PBS. In control experiments, where tissue sections incubated with normal mouse IgG instead of the primary antibody served as the controls, no stained cells were observed in any of sections. The stained specimens were photographed using photomicroscope to evaluate the amount of 8-OHdG in cornea. The reciprocal number of brightness of stained area in corneal picture was defined as the amount of 8-OHdG. The brightness was estimated using Adobe Photoshop CS5 (Adobe systems incorporated, Tokyo Japan).

## Results

### Preparation of Se-apolactoferrin

**Figure 1:**
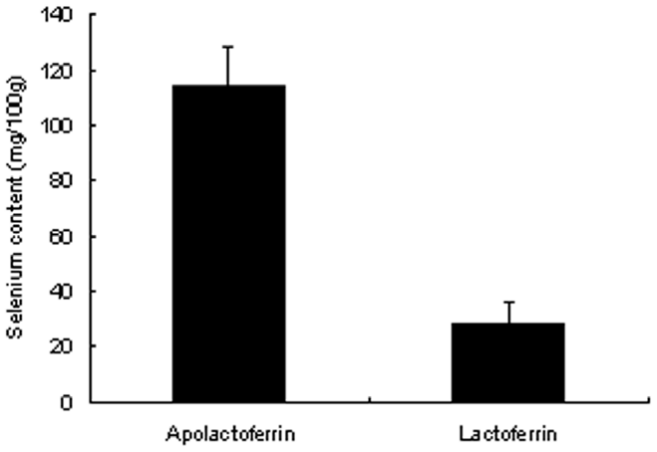
Comparison of capacity of apolactoferrin and lactoferrin for selenium binding .The selenium content in apolactoferrin and normal lactoferrin is shown. The vertical axis shows selenium content (mg) in 100g of apolactoferrin or normal lactoferrin. Results are expressed as mean ± S.D (n = 6).

Apolactoferrin could bind a significantly larger amount of selenium than normal lactoferrin (Fig. 1). It is thought that selenium was less susceptible to replace with iron in lactoferrrin. Although 100 g of normal lactoferrin bound 24.3 mg of selenium, 100 g of apolactoferrin could bind 116.1 mg of selenium.

### Selenium Uptake to Corneal Epithelium Cells

**Figure 2:**
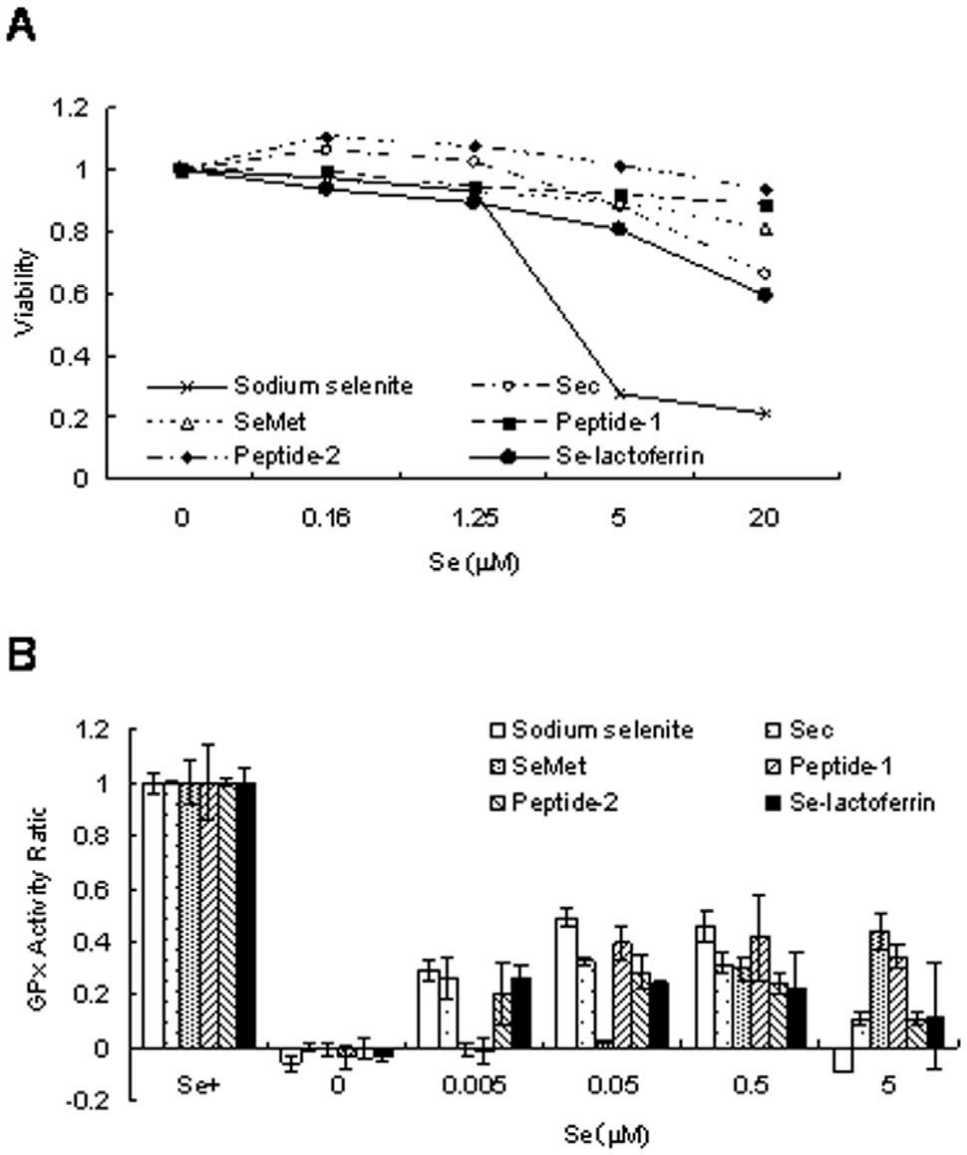
Effect of selenium compounds in CEPI cells. A: Effect of selenium compounds on cellular viability of CEPI cells. The horizontal axis shows the selenium concentration of each selenium compound. B: Recovery of GPx activity by the addition of selenium compounds. The vertical axis shows the GPx activity ratio. Results are expressed as mean ± S.D.

Selenium compounds influenced the cellular viability of CEPI cells (Fig. 2A). Comparing the toxicity of selenium compounds in the absence of Se-lactoferrin, sodium selenite was the most toxic, selenoamino acids were next, and selenopeptides were less toxic compounds. Se-lactoferrin was somewhat toxic compared with selenoamino acids, but better by far than sodium selenite. All selenium compounds were uptaken to CEPI cells; however, their efficiency, which was shown as the GPx activity ratio, and optimum concentration of each compound showed diverse values (Fig. 2B). The maximum uptake efficiency of sodium selenite and Sec occurred at low selenium concentrations, while the maximum uptake efficiency of SeMet occurred at a high concentration. Peptide-1 and Se-lactoferrin were uptaken over a wide range of selenium concentrations. The concentration of selenium compounds in eye drops was decided based on the results in Figure 2.

### Effects of Selenium Compounds on the Treatment of Dry Eye

**Figure 3:**
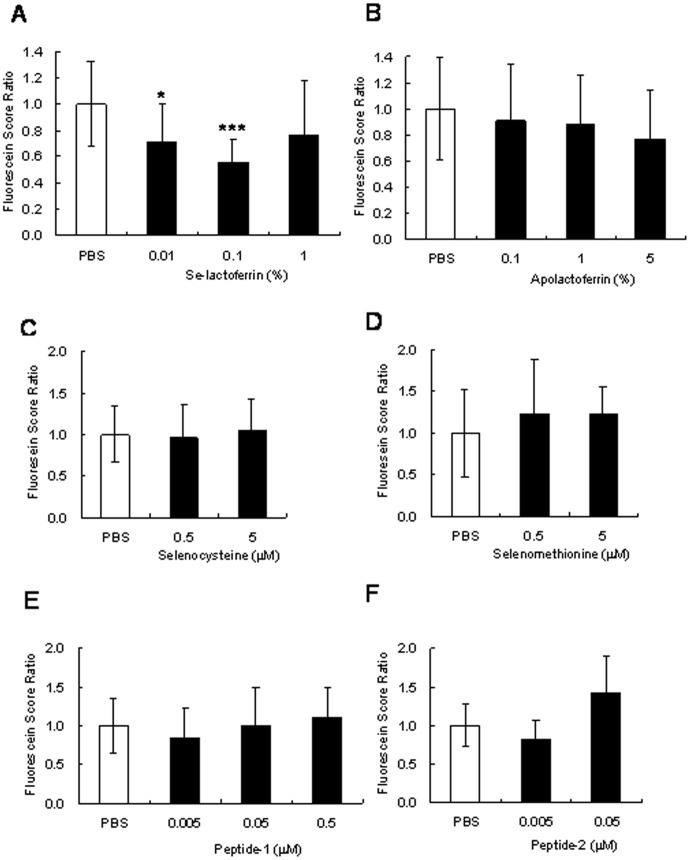
Effects of Selenium compounds eye drops on the cornea of a dry eye rat model. Fluorescein score ratios of cornea in dry eye rat treated with selenium compounds (A, C–F), apolactoferrin (B), or PBS (A–F) or are shown. The score of PBS treatment was defined as 1. Results are expressed as mean ± S.D (n = 10). Dunnett’s test was used to determine the significance of differences. * and *** indicate a significant difference from the result in PBS treatment, P<0.05 and <0.005, respectively (A).

Figure 3 shows the results after administering selenium compound eye drops. The fluorescein score was varied even in normal cornea, because the corneal condition was not uniform owing to their individual difference in rats. Se-lactoferrin eye drops could suppress corneal irritation, especially 0.1% Se-lactoferrin eye drops (Fig. 3A). On the other hand, 5% apolactoferrin eye drops slightly suppressed the increased fluorescein scores (Fig. 3B). Treatment for dry eye was more effective using selenium-containing lactoferrin than apolactoferrin. Figures 3C, 3D, 3E, and 3F show the results after administering other selenium compound eye drops on treatment for dry eye. They did not suppress the increased fluorescein scores except 0.005 µM peptide-2 eye drop. It was concluded that a selenium compound other than Se-lactoferrin is not necessarily useful for the treatment of dry eye.

### Gene Expression in Corneas

**Figure 4:**
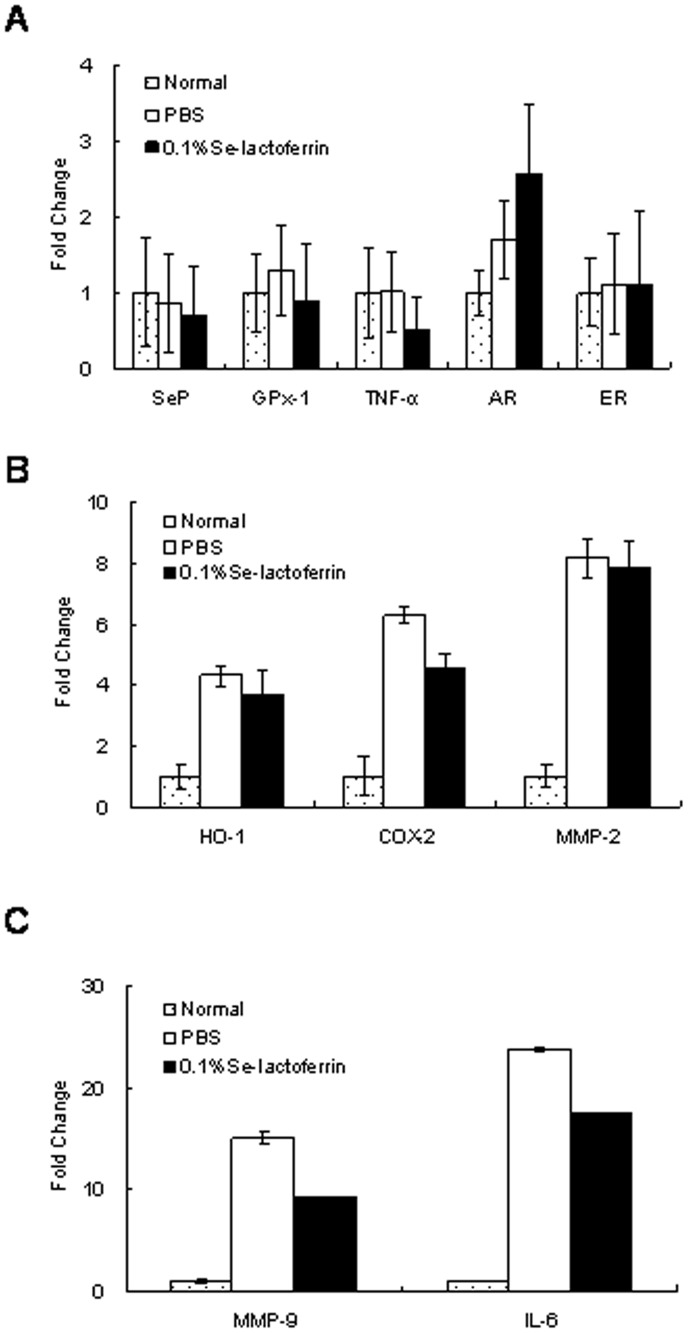
Effect of Se-lactoferrin eye drops on expression of RNAs in the corneas. The vertical axis shows the expression ratio of RNAs. Fold change shows the expression ratio of mRNAs in the NT, PBS, and 0.1%Se-loactoferrin groups. The expression level in the NT group was defined as 1. Results are expressed as the mean ± S.D. (n = 4).

The relative expression level of mRNAs was estimated using the ΔCt value. The ΔCt value was calculated using Ct values in each protein and GAPDH. If the ΔCt value was lower, the relative RNA level was higher. The ΔCt value of SeP, GPx-1, TNF-α AR, ER, HO-1, COX-2, MMP-2, MMP-9, and IL-6 in the NT group was 4.0±0.5, 4.3±1.0, 10.8±1.3, 10.0±1.1, 9.5±1.5, 11.6±2.4, 14.4±0.1, 9.1±0.7, 13.9±1.7, and 12.1±1.1, respectively. Figure 4 shows the fold change of mRNA prepared from the cornea. Expression of HO-1, COX-2, MMP-2, MMP-9, and IL-6 increased markedly in the dry eye group. 0.1% Se-lactoferrin eye drops suppressed the up-regulation of HO-1, COX-2, MMP-9, and IL-6, and slightly suppressed that of MMP-2. AR expression increased by induction of dry eye and further increased by treatment with 0.1% Se-lactoferrin eye drops. TNF-α expression was not changed by induction of dry eye, while it was reduced in dry eye rats treated with 0.1% Se-lactoferrin eye drops. There were no differences in the expression of SeP, GPx-1, and ER among the groups.

### Se-lactoferrin Eye Drops Suppressed 8-OHdG Induction

**Figure 5:**
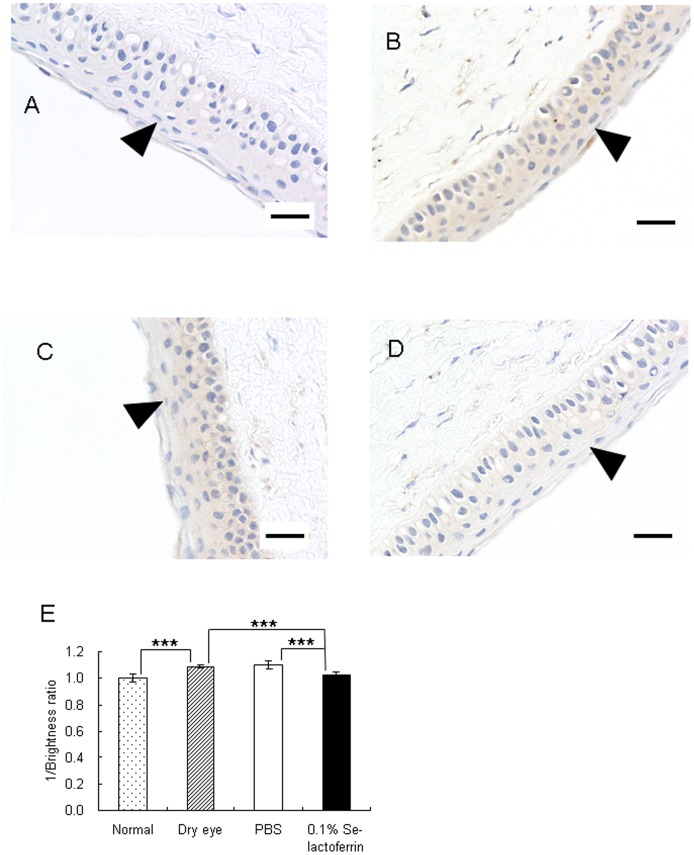
Effect of Se-lactoferrin eye drops on production of 8-OHdG in corneas. A, B: Immunohistochemical staining of corneas of normal (A) and dry eye (B) rats using an anti-8-OHdG antibody. C,D: Immunohistochemical staining of corneas of dry eye rats treated with PBS (C) or 0.1% Se-lactoferrin (D) using anti-8-OHdG antibody. Scale bar shows 20 µm. Original magnification: x20. E: Relative ratio of 8-OHdG in corneas of normal, dry eye, dry eye treated with PBS or dry eye treated with 0.1% Se-lactoferrin rat. The vertical axis shows 1/brightness ratio. The brightness ratio in the normal group was defined as 1. Results are expressed as the mean ± S.D. (n = 4). Dunnett’s test was used to determine the significance of differences. *** indicate a significant difference from the results in normal or PBS treatment group, P<0.005.

Immunohistochemical analysis revealed that 8-OHdG production was increased in the corneal epithelium of dry eye rats (Fig. 5B) compared with that of normal rats (Fig. 5A). PBS eye drops had no effect on 8-OHdG production (Fig. 5C), whereas 0.1% Se-lactoferrin eye drops remarkably suppressed 8-OHdG production (Fig. 5D). These results were digitized in Figure 5E, in which the amount of 8-OHdG in cornea of each group was relatively represented. Increase of 8-OHdG by dry eye treatment was significantly suppressed by Se-lactoferrin eye drops. Se-lactoferrin eye drops were able to suppress production of oxidative stress in the corneal epithelium.

## Disucussion

The ocular surface is exposed to oxidative stress generated by ultraviolet radiation, environmental pollutants, etc. Tear fluid is essential to maintain the functions of the ocular surface including anti-oxidative stress. Dry eye is caused by acceleration of tear evaporation from the ocular surface and reduction of tear secretion due to dysfunction of lacrimal glands accompanied by an increase in oxidative stress in the corneal epithelium [6], [12]. Since selenium is essential for the activity of oxidative stress-regulating enzymes, we expected that providing selenium to the ocular surface would be useful for the treatment of dry eye. Se-lactoferrin eye drops caused notable improvement in corneal damage induced by dry eye, whereas apolactoferrin eye drops did not show a remarkable effect (Fig. 3B). These results supported our expectation. Furthermore, we supposed that the other selenium compounds used in this study would also improve the ocular surface of dry eye, because the selenium in these compounds was uptaken into CEPI cells similar to Se-lactoferrin (Fig. 2B). Contrary to our expectation, not all selenium compounds were effective (Fig. 3). Our previous study showed that SeP eye drops improved corneal damage and SeP is a physiological selenium carrier to the corneal epithelium in tear fluid [12]. Lactoferrin is also a physiological iron carrier to the corneal epithelium [13]. It is supposed that a physiological mechanism, for example, a ligand-receptor system, is essential for selenium uptake to the corneal epithelium in an organism, unlike cultured cells.

In the meantime, apolactoferrin eye drops at high dose showed a weak but not statistically significant improvement in corneal damage. A previous study showed that lactoferrin eye drops rescued the corneal damage using the rabbit dry eye model, and the effect was more remarkable using lactoferrin, which was not saturated with iron in the physiological condition, compared with iron-saturated lactoferrin [24]. Se-lactoferrin eye drops may have two effects, i.e., the effect due to selenium and the effect due to apolactoferrin. The contribution of apolactoferrin, however, was probably small upon using 0.1% Se-lactoferrin eye drops because slight effectiveness was observed using 0.1% apolactoferrin eye drops.

COX-2, MMP-9, and IL-6 expression are elevated in the cornea of dry eye [25], [26]. The results from Figure 4 support that Se-lactoferrin eye drops improved dry eye. Induction of dry eye did not affect TNF-α expression, which is consistent with the results from our previous study [27]. Previous studies showed that corneal functions were regulated by sex hormones through the AR and ER [28]–[30]. The efficacy of Se-lactoferrin is possibly mediated by up-regulation of AR expression. The expression of selenium enzymes, *i.e.*, SeP and GPx-1, in the cornea of dry eye rats treated with Se-lactoferrin did not change (Fig. 4A), whereas CEPI cells could uptake Se-lactoferrin and use it for GPx synthesis (Fig. 2B). It is supposed that GPx synthesis in the cornea is not regulated by transcription of mRNA.

HO-1 expression was increased by induction of dry eye and decreased by Se-lactoferrin eye drops (Fig. 4B). Since HO-1 expression is up-regulated by oxidative stress, Se-lactoferrin eye drops probably suppress the production of oxidative stress. Similar results were obtained from the immunohistochemical analysis (Fig. 5C, 5D). 8-OHdG production increased in the corneal epithelium of dry eye rats compared with that of normal rats (Fig. 5A, 5B). It is supposed that Se-lactoferrin eye drops improve corneal damage in dry eye by suppressing the production of oxidative stress in the cornea. Dry eye syndrome is related to oxidative stress, and selenium compounds are probably candidates for treatment of dry eye by regulating oxidative stress in the corneal epithelium.
